# Learning-based super-resolution technique significantly improves detection of coronary artery stenoses on 1.5T whole-heart coronary MRA

**DOI:** 10.1186/1532-429X-16-S1-P218

**Published:** 2014-01-16

**Authors:** Masaki Ishida, Ryohei Nakayama, Mio Uno, Tatsuro Ito, Yoshitaka Goto, Yasutaka Ichikawa, Motonori Nagata, Kakuya Kitagawa, Shiro Nakamori, Kaoru Dohi, Masaaki Ito, Hajime Sakuma

**Affiliations:** 1 Radiology, Mie University Hospital, Tsu, Mie, Japan; 2 Cardiology, Mie University Hospital, Tsu, Mie, Japan

## Background

**Figure 1:**
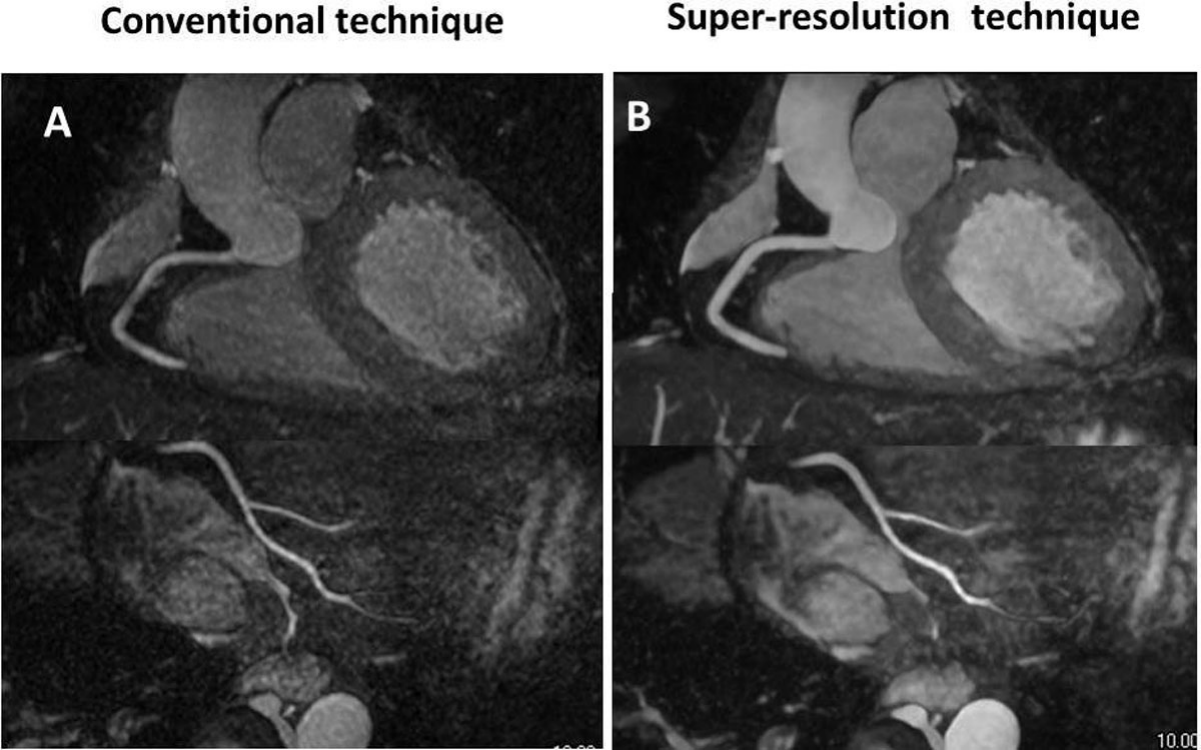
**Example of conventional whole-heart coronary MRA (A) and high-resolution whole-heart coronary MRA with super-resolution technique (B) in the same subject**.

Whole-heart coronary MRA permits noninvasive detection of coronary artery disease. In the brain MRI, super-resolution (SR) technique has emerged as a method to enhance image resolution and improve diagnostic performance. In the current study, we developed a learning-based SR technique optimized for whole-heart coronary MRA (Figure 1). Summarizing briefly, this SR algorithm consists of (1) construction of the dictionary for low- and high-resolution pairs of image patches, and (2) generation of high-resolution coronary MRA images by embedding optimal patches selected form the dictionary by least square fitting. The purpose of this study was to determine if the SR technique can improve the diagnostic performance of 1.5T whole-heart coronary MRA in detecting significant stenosis on catheter coronary angiography.

## Methods

Forty-six patients with suspected coronary artery disease (CAD) underwent X-ray coronary angiography and whole-heart coronary MRA with a 1.5T MR scanner and 32 channel coils. Image quality was assessed using 5-point scale (1 = not visible, 2 = poor, 3 = moderate, 4 = good, 5 = excellent) for conventional coronary MRA. High-resolution coronary MRA was generated by using the SR technique. Three observers independently rated the confidence level of the presence of stenosis in each coronary segment with a continuous scale from 0 to 1 by using a sliding SLAB MIP method. Receiver operating characteristic (ROC) analysis was employed to evaluate the diagnostic performance of high-resolution coronary MRA generated by SR technique in comparison with coronary MRA without SR technique in detecting luminal narrowing of >50% on X-ray angiography.

## Results

**Figure 2:**
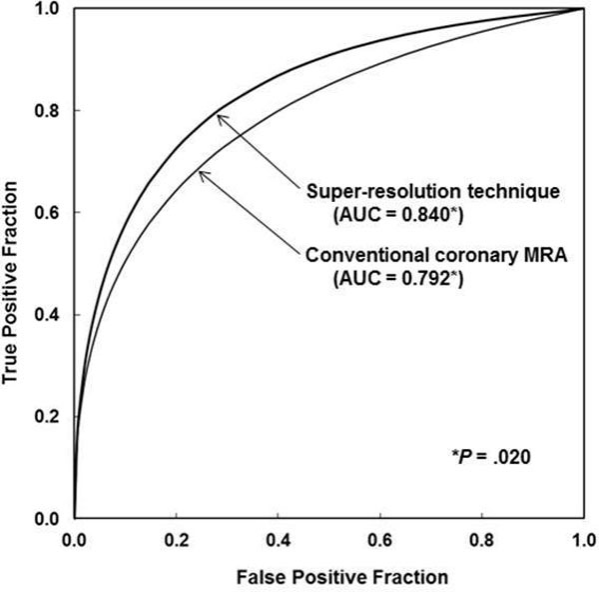
**Comparison of ROC curves for the average performance of the three observers in detection of coronary artery stenoses on high-resolution coronary MRA generated by SR technique and conventional coronary MRA**. With super-resolution technique, the average AUC was significantly improved from 0.792 to 0.840 (p = .020)

By using SR technique, the areas under the ROC curves (AUCs) were improved for all observers. Average AUC was 0.840 for SR technique, being significantly higher than that for conventional coronary MRA (0.792, p = .020) (Figure 2). Intrarclass correlation coefficient among 3 observers was 0.670 by SR technique and 0.579 by conventional coronary MRA, respectively. Mean interpretation time per patient was substantially reduced from 6.6 min to 4.5 min by SR technique. The improvement of mean AUC by SR technique was significant in the segments with higher image quality (mean scale >3) (0.806 to 0.858, p = .023), whereas that was not significant in the segments with lower image quality (mean scale <3) (0.764 to 0.790, p = .562).

## Conclusions

High-resolution whole-heart coronary MRA generated by the SR technique allows for more accurate detection of coronary stenoses with reduced interpretation time as compared to conventional whole-heart coronary MRA.

## Funding

n/a.

